# Endometrial expression of the insulin-like growth factor system during uterine involution in the postpartum dairy cow^☆^

**DOI:** 10.1016/j.domaniend.2007.11.003

**Published:** 2008-05

**Authors:** S. Llewellyn, R. Fitzpatrick, D.A. Kenny, J. Patton, D.C. Wathes

**Affiliations:** a Reproduction, Genes and Development Group, Department of Veterinary Basic Sciences, Royal Veterinary College, Hawkshead Lane, Hatfield, Herts, London AL9 7TA, UK; b Animal Production Research Centre, Mellows Campus, Athenry, Co. Galway, Ireland; c School of Agriculture, Food Science and Veterinary Medicine, University College Dublin, Ireland; d Teagasc Moorepark, Dairy Production Research Centre, Fermoy, Co. Cork, Ireland

**Keywords:** Bovine, Uterus, IGF, IGFBP, Involution

## Abstract

Rapid uterine involution in the postpartum period of dairy cows is important to achieve a short interval to conception. Expression patterns for members of the insulin-like growth factor (IGF) family were determined by *in situ* hybridisation at day 14 ± 0.4 postpartum (*n* = 12 cows) to investigate a potential role for IGFs in modulating uterine involution. Expression in each uterine tissue region was measured as optical density units and data were analysed according to region and horn. IGF-I mRNA was localized to the sub-epithelial stroma (SES) of inter-caruncular and caruncular endometrium. Both IGF-II and IGF-1R expression was detected in the deep endometrial stroma (DES), the caruncular stroma and myometrium. IGFBP-2, IGFBP-4 and IGFBP-6 mRNAs were all localised to the SES of inter-caruncular and caruncular uterine tissue, and in the DES and caruncular stroma, with IGFBP-4 mRNA additionally expressed in myometrium. IGFBP-3 mRNA was only detectable in luminal epithelium. IGFBP-5 mRNA was found in myometrium, inter-caruncular and caruncular SES and caruncular stroma. These data support a role for IGF-I and IGF-II in the extensive tissue remodelling and repair which the postpartum uterus undergoes to return to its non-pregnant state. The differential expression of binding proteins between tissues (IGFBP-3 in epithelium, IGFBP-2, -4, -5 and -6 in stroma and IGFBP-4 and -5 in myometrium) suggest tight control of IGF activity within each compartment. Differential expression of many members of the IGF family between the significantly larger previously gravid horn and the previously non-gravid horn may relate to differences in their rate of tissue remodelling.

## 1 Introduction

In dairy cows, the peri-partum period is critical to future milk production and fertility. Uterine involution involves extensive restructuring of the extracellular matrix alongside mitogenesis and apoptosis [1–3]. Initial degeneration of placental cotyledons and maternal caruncles accumulate as tissue debris in the uterine lumen forming a lochial discharge [4]. Contractions of the myometrium aid expulsion of lochia, and also restore uterine size, shape and tone to that of a non-pregnant animal [5,6]. Whilst most of these changes have occurred within 2–3 weeks postpartum, involution is not considered complete until about 40–50 days postpartum [1]. The previously non-gravid uterine horn returns to a non-pregnant state 10–15 days earlier than the previously gravid uterine horn [7]. Histological repair of the endometrium lags physical involution by 10–20 days [8], completing when caruncles regenerate epithelium [4]. Microbial contamination of the postpartum uterus is almost universal during the first week postpartum [9]. When pathogenic bacteria are not cleared the uterus becomes infected and inflamed and uterine involution is delayed [1,10]. Clinical endometritis is characterised by the continued presence of a purulent discharge beyond 21 days after calving [1].

Many processes involved in uterine repair are common to those of wound healing in other tissues (for a review see [11]). Potential mediators of tissue turnover and remodelling in the uterus include cytokines, matrix-degrading enzymes and growth factors [11,12]. The insulin-like growth factors (IGF-I and IGF-II) function in such tissue repair processes. In healing-impaired wounds, the mRNA for IGF-I, IGF-1R, and IGFBP-3 is significantly reduced [13]. The administration of IGF-I to these wounds corrects defective tissue repair [14] and in combination with other growth factors it increases connective tissue regeneration and epithelialisation [15]. Components of the IGF system have been described in the uteri of a variety of species (e.g. humans [16], rodents [17], pigs [18], cattle [19], and sheep [20]). The proliferative and differentiating effects of IGFs on uterine cells are thought to support the growth and regression of uterine tissue throughout the estrous cycle and also the regenerative processes in women following menstruation [16,21]. IGFBP-2 has also been shown to stimulate endometrial cell mitogenesis directly [22].

An increased rate of uterine involution is associated with earlier resumption of ovarian activity [23], which is in turn important for increasing pregnancy rate to first service [24]. Conversely, endometrial damage associated with sub-clinical endometritis leads to prolonged intervals to conception, with many cows failing to conceive at all [25]. The mechanisms that regulate uterine involution are not completely understood and, to the best of our knowledge, no previous studies have investigated the uterine IGF system during involution in lactating dairy cows. We postulated that changes in IGF bioavailability may be implicated in the rate of postpartum uterine recovery and thus influence the calving to conception interval and reproductive efficiency. The objective of the study was to determine patterns of mRNA expression for the IGF system within the previously gravid (PG) and previously non-gravid (PNG) uterine horns during the early postpartum period. Samples were obtained at approximately 2 weeks after calving as we hypothesised that this represents a time by which a delay in the normal recovery process may predispose cows to the subsequent development of endometritis.

## 2 Materials and methods

### 2.1 Animals and tissue samples

All procedures were carried out under license in accordance with the European Community Directive, 86-609-EC. Uteri were collected from 12 multiparous Holstein-Friesian dairy cows (mean parity 4.7) following slaughter at day 14 ± 0.4 postpartum. The diameters of both horns were measured approximately 5 cm anterior to the bifurcation of the uterus. Samples of inter-caruncular and caruncular tissue were dissected from the previously gravid and non-gravid uterine horns approximately 1 cm anterior to the bifurcation of the uterus. A 5 cm square region was harvested, wrapped in aluminium foil, and frozen in liquid nitrogen-tempered isopentane. Samples were stored at −80 °C until sectioning.

### 2.2 *In situ* hybridisation

The *in situ* hybridisation procedure was performed as described previously [26]. All chemicals were purchased from Sigma–Aldrich Company Ltd. (Poole, Dorset, UK) or VWR International Ltd. (Poole, Dorset, UK) unless otherwise specified. Briefly, sections of 10 μm were cut from each uterine tissue sample and thaw-mounted onto SuperFrost^®^ Plus or POLYSINE™ microscope slides, fixed in 4% (w/v) paraformaldehyde in 0.01 M PBS, washed in PBS, and sequentially dehydrated in 70% and 95% ethanol. The oligonucleotide probes for the IGF system were end-labelled with [^35^S]dATP (Amersham Biosciences UK Ltd., Buckinghamshire, England) using terminal deoxynucleotidyl transferase (Promega UK Ltd., Southampton, England). Tissue sections were subsequently treated with 100 000 cpm (100 μl)^−1^ hybridisation buffer and hybridised overnight at either 42, 45, or 52 °C (Table 1). Following incubation, slides were washed in a solution of 1 × SSC, 2 g l^−1^ sodium thiosulphate at room temperature for 30 min followed by fresh 1 × SSC, 2 g l^−1^ sodium thiosulphate at 60 °C for 60 min. Slides were then rinsed in solutions of 1 × SSC, 0.1 × SSC, 75% ethanol and 95% ethanol and air-dried before exposure to β-max hyperfilm (Kodak BioMax MR Film) for either 4 or 5 days. All uterine sections treated with a particular probe were hybridized in the same batch. Sense probes, which were identical in sequence to the respective mRNA targets, were always included as negative controls and any signal from these was regarded as non-specific. Each batch also contained an appropriate positive control tissue, based on previous studies. These were cross-sections of uterus from an estrous ewe for IGF-I and the type 1 IGF receptor [20], IGFBP-1 [27] and IGFBP-6 [28]; ovine placentome for IGF-II and IGFBPs-2, -3 and -4 [29] and ovine intercotyledonary tissue for IGFBP-5 [30].

### 2.3 Photographic emulsions

To aid cellular localisation of hybridised probes, slides previously subject to autoradiography were coated with photographic emulsion LM1 (Amersham Biosciences UK Ltd., Buckinghamshire, England) according to the manufacturer's instructions and stored for 28, 30 or 42 days at 4 °C in the dark (Table 1). The slides were developed in 20% phenisol (ILFORD Imaging UK Ltd., Cheshire, England) fixed in 1.9 M sodium thiosulphate and counterstained with haematoxylin and eosin. All other slides were also stained with haematoxylin and eosin to aid identification of tissue region.

### 2.4 Optical density measurements

Readings were obtained from at least two sections per tissue for each of the antisense (AS) and sense (S) probes. Autoradiographs were scanned into a computer and optical density (OD) measurements were recorded from digital images. The relative expression of mRNA for components of the uterine IGF system was quantified from the autoradiographs using the public domain NIH ImageJ program (available through the NIH website—http://www.nih.gov), which calculated the average optical density (OD) over the selected area of film based on a linear grey scale of 0.01–2.71. The following tissue layers were each assessed separately: luminal epithelium, sub-epithelial stroma (a layer of dense connective tissue underlying the luminal epithelium), caruncular stroma (the dense connective tissue forming the caruncles), deep endometrial stroma (loose connective tissue between the sub-epithelial stroma and the myometrium) and myometrium. The latter two tissue types were only present in samples collected from the inter-caruncular region. Each tissue type was measured separately on each section. The background OD, from a blank area of film, was also measured and subtracted from both AS and S OD measurements. Finally the S values were subtracted from AS values to give an average OD value for specific hybridisation [31]. The detection limit was taken as an OD value of 0.01.

### 2.5 Statistical analysis

Statistical analyses were performed using Statistical Package for the Social Sciences (SPSS for Windows, V13.0). Data for uterine diameter measurements at the time of tissue collection were analysed using Student's *t*-test. OD measurements were obtained from four samples per cow, taken from each of the caruncular and inter-caruncular regions of the previously gravid and non-gravid horns. The effects of uterine horn and tissue region on the level of mRNA expression for each probe were analysed by general linear model analysis. Cow was entered as a random effect. For this purpose, data from uteri in which a particular probe showed no detectable specific hybridisation (OD of <0.01) were given an OD of 0.01, which equated to the lower limit of detection. Results were considered statistically significant when *P* < 0.05.

## 3 Results

**Fig. 1:**
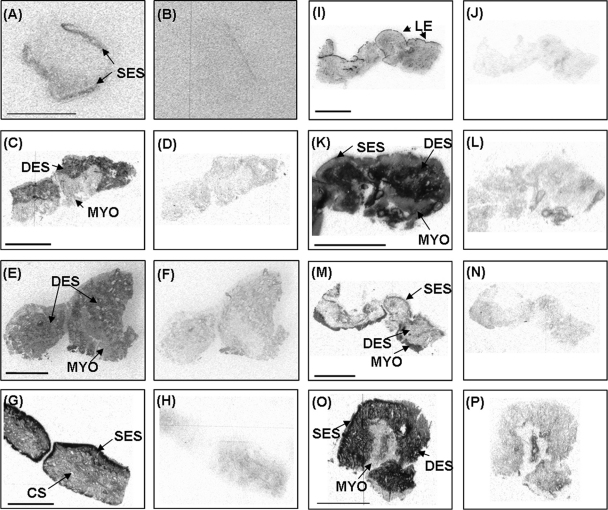
Expression of mRNA for the IGF system in the involuting uterus of postpartum dairy cows. Autoradiographic localisation of mRNA for: (A and B) IGF-I, (C and D) IGF-II, (E and F) IGF-1R, (G and H) IGFBP-2, (I and J) IGFBP-3, (K and L) IGFBP-4, (M and N) IGFBP-5, and (O and P) IGFBP-6. Examples of antisense (A, C, E, G, I, K, M and O) and sense (B, D, F, H, J, L, N and P) probes are illustrated. IGF-I mRNA was expressed in the sub-epithelial stroma (SES); IGF-II and IGF-1R mRNA was detected in the deep endometrial stroma (DES), caruncular stroma (not shown) and myometrium (MYO). IGFBP-2 mRNA was detected in the SES, DES and CS. The expression of IGFBP-3 mRNA was confined to the luminal epithelium (LE). IGFBP-4, IGFBP-5 and IGFBP-6 mRNA was localised to the SES and CS with IGFBP-4 and IGFBP-6 mRNA additionally detected in DES. IGFBP-4 and IGFBP-5 mRNA was also expressed in the MYO. Scale bars = 5 mm.

**Fig. 2:**
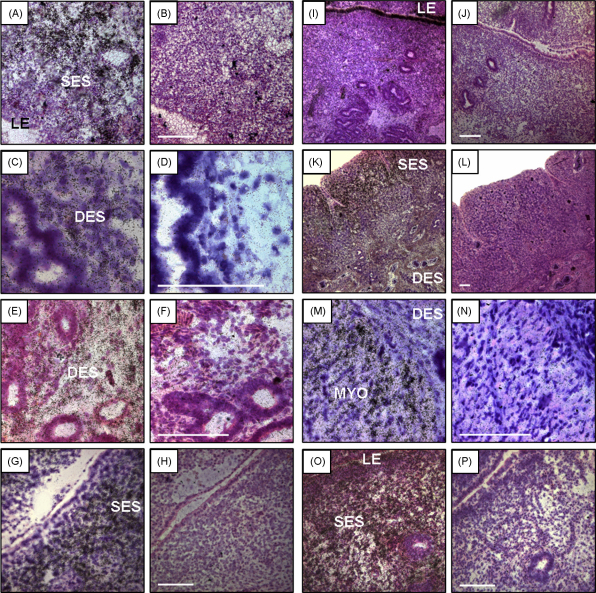
Photographs of uterine sections coated with photographic emulsion and counterstained with haematoxylin and eosin to determine the cellular localisation of mRNA for (A and B) IGF-I, (C and D) IGF-II, (E and F) IGF-1R, (G and H) IGFBP-2, (I and J) IGFBP-3, (K and L) IGFBP-4, (M and N) IGFBP-5, and (O and P) IGFBP-6. Examples of antisense (A, C, E, G, I, K, M and O) and sense (B, D, F, H, J, L, N and P) probes are illustrated. SES, sub-epithelial stroma; DES, deep endometrial stroma; LE, luminal epithelium; MYO, myometrium. Scale bars = 100 μm.

**Fig. 3:**
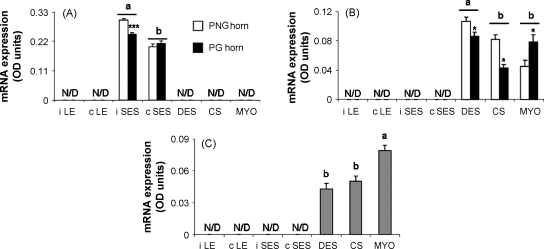
Expression of (A) IGF-I, (B) IGF-II, and (C) IGF-1R mRNA in different tissue regions and uterine horns of the involuting bovine uterus. iLE, inter-caruncular luminal epithelium; cLE caruncular luminal epithelium; iSES, inter-caruncular sub-epithelial stroma; cSES, caruncular sub-epithelial stroma; DES, deep endometrial stroma; CS, caruncular stroma; MYO, myometrium; PNG, previously non-gravid horn; PG, previously gravid horn. Values are mean ± S.E.M. of data from 12 cows. Differences due to tissue region are indicated with different superscript letters (*P* ≤ 0.001). Differences due to horn for a particular tissue region are indicated by ^*^P < 0.05, ^***^P ≤ 0.001.

**Fig. 4:**
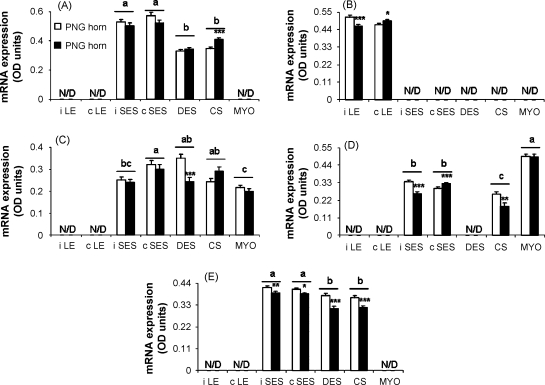
Expression of (A) IGFBP-2, (B) IGFBP-3, (C) IGFBP-4, (D) IGFBP-5, and (E) IGFBP-6 mRNA in different tissue regions and uterine horns of the involuting bovine uterus. iLE, inter-caruncular luminal epithelium; cLE caruncular luminal epithelium; iSES, inter-caruncular sub-epithelial stroma; cSES, caruncular sub-epithelial stroma; DES, deep endometrial stroma; CS, caruncular stroma; MYO, myometrium; PNG, previously non-gravid horn; PG, previously gravid horn. Values are mean ± S.E.M. of data from 12 cows. Differences due to tissue region are indicated with different superscript letters (*P* ≤ 0.001). Differences due to horn for a particular tissue region are indicated by ^*^P < 0.05, ^**^P < 0.010, and ^***^P ≤ 0.001.

At the time of tissue collection, the diameter of the previously gravid uterine horn was larger than that of the previously non-gravid uterine horn (56 ± 6.9 and 31 ± 3.1 mm, respectively, mean ± S.E.M., *P* = 0.005). The spatial distribution of mRNA encoding components of the uterine IGF system is shown in Figs. 1 and 2. The concentrations of mRNA in OD units are summarised in Table 2 according to uterine horn and tissue region and their two-way interactions are illustrated in Figs. 3 and 4. The method used provided a semi quantitative measure of the intensity of mRNA expression in specific cell types.

### 3.1 Expression of the IGFs and IGF type 1 receptor

IGF-I mRNA was localized to the sub-epithelial stroma (SES) of inter-caruncular and caruncular endometrium in both uterine horns (Figs. 1(A) and 2A). Both IGF-II and IGF-1R expression was detected in the deep endometrial stroma (DES), the caruncular stroma (not shown) and myometrium (Figs. 1(C), (E) and 2C, E).

Overall expression of IGF-I mRNA was higher in the inter-caruncular than caruncular SES (*P* = 0.001, Table 2). There was a significant horn × region interaction (*P* = 0.032), with lower levels of IGF-I transcript in the inter-caruncular SES of the PG compared with the PNG horn (Fig. 3(A)). IGF-II expression was higher in the DES than in the caruncular stroma and myometrium (*P* ≤ 0.001, Table 2). When data from tissue regions were pooled the concentration of IGF-II mRNA did not vary between the PNG and PG horns, but there was a significant horn × region interaction (*P* ≤ 0.001). Expression of IGF-II in the DES and caruncular stroma was lower in the PG than PNG horn, whereas within myometrium expression was higher in the PG than PNG horn (Fig. 3(B)). For the IGF-1R, expression was highest in myometrium and similar between DES and caruncular stroma (Table 2 and Fig. 3(C)). Overall, the level of IGF-1R transcript was higher (*P* = 0.030) in the PNG than PG horn (Table 2). The horn × region interaction was not significant for uterine IGF-1R mRNA expression.

### 3.2 Expression of IGFBPs

IGFBP-1 mRNA could not be detected in any uteri examined, despite expression being observed in the ovine estrous uterus which was used as positive control tissue (data not shown). IGFBP-2, IGFBP-4 and IGFBP-6 mRNAs were all localised to the SES of inter-caruncular and caruncular uterine tissue, and in the DES and caruncular stroma (Figs. 1(G), (K), (O) and 2G, K, O). IGFBP-4 mRNA was additionally expressed in myometrium. In contrast, IGFBP-3 mRNA expression was only detected in the luminal epithelium (LE) of both inter-caruncular and caruncular samples (Figs. 1(I) and 2I). IGFBP-5 mRNA was found in myometrium, inter-caruncular and caruncular SES and caruncular stroma (Figs. 1(M) and 2M).

IGFBP-2 mRNA expression in inter-caruncular and caruncular SES was higher than in DES and caruncular stroma (*P* ≤ 0.001, Table 2). There was no main effect of horn, but there was a horn × region interaction (*P* = 0.034). Within caruncular stroma only, the concentration of IGFBP-2 mRNA was higher in the PG than the PNG uterine horn (Fig. 4(A)).

For IGFBP-3 mRNA the main effects of uterine horn and tissue region were not significant but there was an interaction (*P* ≤ 0.001). Expression in the inter-caruncular LE was higher in the PNG than PG horn, whereas in the caruncular LE expression was higher in the PG uterine horn (Fig. 4(B)).

Expression levels of IGFBP-4 mRNA varied between tissue regions, with higher expression in the caruncular than inter-caruncular SES, lowest expression in myometrium, and intermediate signal intensity in the DES and caruncular stroma (Table 2). There was no difference in transcript levels between the PNG and PG uterine horns when regional data were combined (Table 2). Levels of IGFBP-4 mRNA expression were, however, affected by an interaction between uterine horn and tissue region (*P* = 0.024): within DES expression was lower in the PG than PNG uterine horn (Fig. 4(C)).

Expression of IGFBP-5 mRNA was highest in myometrium, intermediate in the inter-caruncular and caruncular SES and lowest in caruncular stroma (*P* ≤ 0.001, Table 2). When regional expression data were pooled, the PNG uterine horn expressed higher concentrations of IGFBP-5 mRNA than the PG horn (*P* ≤ 0.001, Table 2). There was also a significant effect of the interaction between uterine horn and tissue region (*P* ≤ 0.001). Expression in both the inter-caruncular SES and the caruncular stroma was lower in the PG than PNG uterine horn whereas for the caruncular SES the reverse was true (Fig. 4(D)).

IGFBP-6 mRNA was expressed at higher concentrations in the inter-caruncular and caruncular SES than in DES and caruncular stroma (*P* ≤ 0.001, Table 2). Transcript levels were higher (*P* ≤ 0.001) in the PNG than PG uterine horn when regional expression data were pooled (Table 2). The interaction between uterine horn and tissue region was significant (*P* = 0.045). Expression in each of the inter-caruncular SES, caruncular SES, DES and caruncular stroma was lower in the PG than PNG horn (Fig. 4(E)).

## 4 Discussion

The rate of uterine involution is an important factor influencing the subsequent fertility of dairy cows [24]. In this study we have investigated for the first time a possible role for the IGF family of proteins in this event in lactating dairy cows. The timing of tissue collection at approximately day 14 postpartum occurred when the PG uterine horn in our group of multiparous cows was larger than the PNG horn. At this stage caruncular tissue is expected to have undergone degeneration and sloughing, but not to have completed re-epithelialisation [2]. In contrast, the inter-caruncular area does not lose its epithelial layer [32] and recovers from pregnancy more quickly [2]. The ongoing process of uterine involution at the time of tissue collection was thus expected to involve tissue regeneration alongside size recovery. An adequate recovery process may be crucial in preventing the uterus, which is heavily contaminated with bacteria following calving [1], from developing endometritis. Samples were analysed using *in situ* hybridisation. Whilst this approach is considered only semi-quantitative, we have found the technique described here to be highly repeatable. Furthermore, it enables measurement of mRNA concentrations in individual cellular types. This is not feasible in a complex organ such as the uterus using alternative techniques such as RT-PCR, as it is not readily possible to separate different populations of epithelial and stromal cells for RNA extraction.

IGF-I mRNA was localised to the SES, confirming earlier observations in the cow [33] and sheep [20,34]. Normal wound healing involves a sequence of inflammation, proliferation, and maturation or remodelling [12] and local IGF-I production increases as wound healing progresses [35]. Since IGF-I increases during the late proliferative phase of the human menstrual cycle [36], and is known to stimulate cell proliferation and collagen synthesis during tissue regeneration [14,35], we propose that IGF-I produced by SES may act in an autocrine and/or paracrine manner to stimulate the proliferation of uterine stroma and epithelium [37,38] during uterine involution.

In early pregnancy the bovine endometrium synthesises IGF-II primarily within caruncular stroma [33]. The present study localised IGF-II mRNA at similar concentrations in both the caruncular stroma and myometrium. The strongest expression of IGF-II mRNA was, however, in the DES. Similar results were found in human endometrium [36]. IGF-1R was similarly localised to the DES, caruncular stroma and myometrium, confirming earlier observations in the bovine uterus [33]. Since the effects of IGF-II are probably mediated by the IGF-1R (for a review see [39]), the co-localisation of IGF-II and IGF-1R transcripts supports a local action for IGF-II in uterine repair and regeneration within both endometrial and caruncular stroma [16,21]. Stromal IGF-II may also act in a paracrine manner to stimulate epithelial cell proliferation [22]. The interaction between IGFs and their receptors in muscle growth and regeneration has been comprehensively reviewed by Florini et al. [40]. In myometrium, IGF-II may stimulate muscle growth and regeneration [40], and potentially increases muscle strength [41]. These actions would support myometrial contractions that return the uterus to its non-pregnant size, shape and tone [5]. Since the PG horn has to contract from a larger size at parturition, the higher concentration of IGF-II in the PG myometrium supports the proposal that IGF-II assists postpartum uterine size recovery. In rat myometrium the IGF-1R is up-regulated in the early postpartum period [42].

This study failed to detect IGFBP-1 mRNA in any postpartum uteri. During the estrous cycle IGFBP-1 expression is low at estrous and relatively higher during the luteal phase [33] concurrent with progesterone production. Since the cows in this study had yet to establish ovulatory cycles, then the uterus would not have been recently exposed to progesterone stimulation [43]. In ruminants IGFBP-1 appears to be involved in pregnancy recognition [33,44] rather than, as this study shows, postpartum uterine events.

In agreement with previous studies [33,44], the expression of uterine IGFBP-2 mRNA was localised to the SES and at relatively lower levels in the DES and caruncular stroma. In the cyclic cow IGFBP-2 mRNA levels increased during the luteal phase, concomitant with the highest levels of plasma progesterone [19]. Other studies have shown that human endometrial cells constitutively synthesise and secrete IGFBP-2 *in vitro* [45] and in response to estradiol [46]. In the bovine mammary gland [47], IGF-I may stimulate IGFBP-2 expression and protein secretion, whereas in fetal visceral glomerular epithelial cells isolated from human kidneys IGFBP-2 production was stimulated by IGF-II [48]. The exact mechanisms regulating postpartum uterine IGFBP-2 mRNA expression thus requires further investigation. IGFBP-2 is presumably modulating uterine involution indirectly by regulating the bioavailability of IGF-I and IGF-II and the interaction of these ligands with their receptors [39]. The precise action of IGFBP-2 also remains uncertain. Both stimulatory [22] and inhibitory [48] actions of IGFBP-2 on IGF-stimulated epithelial cell proliferation have been suggested. Alternatively or additionally, IGFBP-2 could modulate uterine cell growth directly [22].

In contrast to all the other binding proteins investigated, the expression of IGFBP-3 mRNA was confined to the luminal epithelium, again agreeing with earlier work in the cyclic animal [33]. Epithelial IGFBP-3 may regulate local IGF bioavailability [39] or transport IGFs across this cell layer [49,50] for secretion into the uterine lumen. Removing excess IGF from the endometrium would prevent the IGF-1R from being down-regulated [51]. Alternatively, since IGFBP-3 associates with cell surfaces, then it may store IGFs [52] in the uterus and further promote IGF-stimulated tissue repair [37,39] as proposed for other physiological systems [53].

IGFBP-4 mRNA was detected in multiple uterine tissue compartments. The localisation in SES and caruncular stroma has also been found in the pregnant ewe [54] and synthesis in the DES and myometrium agrees with studies in the human and pregnant bovine uterus [44,55]. IGFBP-4 is generally considered inhibitory to IGF actions [56]. IGFBP-4 does not appear to bind to the cell surface or extracellular matrix, but can cross the endothelium [39], indicating that IGFBP-4 may clear endometrial IGFs.

IGFBP-5 mRNA was localised to caruncular stroma and myometrium, in agreement with previous studies in the cow [33] and sheep [34], and transcript was also detected in the SES. With a lack of detectable IGFBP-2, IGFBP-3, and IGFBP-6 mRNA alongside relatively low levels of IGFBP-4 mRNA in the myometrium, the abundance of IGFBP-5 in this tissue suggests this binding protein as the primary regulator of local IGF bioavailability [57]. In the rat myometrium, IGFBP-5 mRNA is significantly up-regulated after parturition, which is suggested to support tissue remodelling during involution [42]. It is also possible that within the myometrium, IGFBP-5 is directly stimulating muscle cell survival during myogenesis [57,58]. In stromal fibroblasts, IGFBP-5 can adhere to the extracellular matrix, which decreases its affinity for IGF and can potentiate IGF-stimulated DNA synthesis [39,59]. Furthermore, IGFBP-5 may stimulate local tissue growth independently of IGF [39,59].

IGFBP-6 was localised to SES and at lower levels in DES and caruncular stroma, similar to the non-pregnant ovine uterus [28]. This expression pattern parallels that of IGFBP-2 mRNA and may suggest these two binding proteins are co-regulated [60]. IGFBP-6 has a markedly higher affinity for IGF-II [61] and so the major function of IGFBP-6 is probably to regulate IGF-II actions [62,63]. Furthermore, IGFBP-6 is generally considered to inhibit the effects of IGF-II, including cell proliferation and differentiation [59]. The importance of controlling uterine IGF bioactivity has been demonstrated in the human uterus where low levels of IGFBP-6 and higher levels of IGF-II are associated with uterine leiomyomas (fibroids), compared with normal endometrium [64].

Many members of the IGF family showed differential expression between the two uterine horns. Expression of IGF-I, the IGF-1R and IGFBP-6 was at an overall lower level in the previously gravid horn whereas IGFBP-4 mRNA expression was lower in the DES only. IGF-II, IGFBP-3 and IGFBP-5 expression showed horn by region interactions, with mRNA concentrations reduced for some regions but increased for others. These differences may reflect the temporal misalignment of the horns in their rate of tissue remodelling, with the PG horn lagging behind the PNG horn by up to 15 days [7].

In conclusion, the IGF system is significant to uterine function and the synthesis of these growth factors in the postpartum uterus indicates a role in uterine involution. This study has shown that in the postpartum bovine uterus IGF-I synthesis was localised to sub-epithelial stroma, whilst maximum concentrations of IGF-II and IGF-1R mRNA were in the endometrial stroma and myometrium, respectively. The uterine tissue compartments expressed different profiles of IGF-binding proteins, indicating that IGF bioavailability and bioactivity is differentially regulated throughout the regenerating endometrium. IGFs are presumably supporting the tissue repair that follows parturition, similar to that of normal wound healing [12]. We propose that myometrial IGF-II synthesis stimulates tissue recovery in an autocrine manner, which may assist the uterus returning to its non-pregnant shape and size. Although IGFs may be key physiological mediators of endometrial repair, other growth factors and cytokines are undoubtedly also important in this process [11,65].

## Conflicts of interest

The authors declare that there is no conflict of interest that would prejudice the impartiality of this scientific work.

## Figures and Tables

**Table 1:** The sense sequences of oligonucleotide probes used for *in situ* hybridisation analysis, their similarity to the equivalent bovine genome and the exposure times for X-ray films and photographic emulsions

Probe	Sense sequence	GenBank accession	Hybridisation temperature (°C)	Exposure time (days)	
				X-ray film	Emulsions
IGF-I	5′-TCACATCCTCCTCGCATCTCTTCTATCTGGCCCTGTGCTTGCTCG-3′	NM_001077828.1	45	7	42
IGF-II	5′-CCAGCGAGACTCTGTGCGGCGGGGAGCTGGTGGACACCCTCCAGT-3′	NM_174087.3	52	4	30
IGF-1R^a^	5′-CTCACGGTCATCCGCGGCTGGAAACTCTTCTACAACTACGCCCTG-3′	XM_871496.2	42	4	42
IGFBP-1	5′-GGAGAGCCTGGGCTCTGTTGGTGTGTCTACCCTTGGAGTGGGAAG-3′	NM_174554.2	45	5	b
IGFBP-2^a^	5′-GCGCCAGCCCCGAGCAGGTTGCAGACAATGGCGAGGAGCACTCTG-3′	NM_174555.1	45	4	42
IGFBP-3	5′-GAGTCGGAAGAAGACCACAGCATGGGGAGCACAGAGAACCAGGCT-3′	NM_174556.1	45	4	28
IGFBP-4	5′-AAGACGGGAGTGAAGCTTCCGGGGGGCCTGGAGCCGAAGGGGGAG-3′	NM_174557.2	45	5	42
IGFBP-5	5′-CTACTCGCCCAAGATCTTCCGGCCCAAGCACACCCGCATCTCCGA-3′	S52657.1	42	4	28
IGFBP-6	5′-CTCTACGTGCCTAATTGTGACCATAGGGGCTTCTACCGGAAGCGG-3′	NM_001040495.1	42	5	42

a The sequence is 97% homologous with the predicted IGF-1R bovine transcript and the bovine IGFBP-2 transcript.

b Autoradiography revealed no hybridisation so emulsions were not prepared.

**Table 2:** Expression of the IGF system by *in situ* hybridisation in the involuting bovine uterus according to uterine horn and tissue region*

	Horn		Region							*P*	
	PNG	PG	Inter-caruncular LE	Caruncular LE	Inter-caruncular SES	Caruncular SES	DES	CS	MYO	Horn	Region
IGF-I	0.26 ± 0.008a	0.23 ± 0.008b	N/D	N/D	0.27 ± 0.008x	0.22 ± 0.008y	N/D	N/D	N/D	**0.012**	≤**0.001**
IGF-II	0.08 ± 0.005	0.07 ± 0.004	N/D	N/D	N/D	N/D	0.10 ± 0.005x	0.06 ± 0.006y	0.06 ± 0.005y	0.108	≤**0.001**
IGF-1R	0.06 ± 0.003a	0.05 ± 0.003b	N/D	N/D	N/D	N/D	0.04 ± 0.003y	0.05 ± 0.003y	0.80 ± 0.003x	**0.030**	≤**0.001**
IGFBP-1	N/D	N/D	N/D	N/D	N/D	N/D	N/D	N/D	N/D	N/D	N/D
IGFBP-2	0.44 ± 0.008	0.45 ± 0.009	N/D	N/D	0.52 ± 0.012x	0.54 ± 0.012x	0.35 ± 0.013y	0.38 ± 0.013y	N/D	0.424	≤**0.001**
IGFBP-3	0.48 ± 0.007	0.49 ± 0.008	0.49 ± 0.008	0.48 ± 0.007	N/D	N/D	N/D	N/D	N/D	0.529	0.697
IGFBP-4	0.28 ± 0.008	0.25 ± 0.008	N/D	N/D	0.25 ± 0.012yz	0.31 ± 0.013x	0.30 ± 0.014xy	0.27 ± 0.014xy	0.21 ± 0.016z	0.105	≤**0.001**
IGFBP-5	0.35 ± 0.007a	0.31 ± 0.007b	N/D	N/D	0.30 ± 0.008y	0.31 ± 0.008y	N/D	0.21 ± 0.012z	0.50 ± 0.012x	≤**0.001**	≤**0.001**
IGFBP-6	0.40 ± 0.005a	0.35 ± 0.005b	N/D	N/D	0.40 ± 0.006x	0.40 ± 0.006x	0.34 ± 0.007y	0.34 ± 0.007y	N/D	≤**0.001**	≤**0.001**

Within rows, values with different letters are significantly different: (a and b) between uterine horns; (x, y, z) between uterine tissue regions.

* Values are mean ± S.E.M. optical density units analysed by general linear model; *n* = 12 cows. Effects that are statistically significant (*P* < 0.05, <0.01 or ≤0.001) are indicated in bold. PNG, previously non-gravid uterine horn; PG, previously gravid uterine horn; LE, luminal epithelium; SES, sub-epithelial stroma; DES, deep endometrial stroma; CS, caruncular stroma; MYO, myometrium; N/D, not detectable in all samples.
